# Sulfide Stress Cracking Behavior of a Martensitic Steel Controlled by Tempering Temperature

**DOI:** 10.3390/ma11030412

**Published:** 2018-03-09

**Authors:** Yu Sun, Qian Wang, Shunjie Gu, Zaoneng He, Qingfeng Wang, Fucheng Zhang

**Affiliations:** 1 Laboratory of Metastable Materials Science and Technology, Yanshan University, Qinhuangdao 066004, China; sunyuysu@163.com (Y.S.); wq986086441@139.com (Q.W.); g897559203@126.com (S.G.); hzncrchi@163.com (Z.H.); zfcysu@163.com (F.Z.); 2 Technical Center, Tianjin Pipe (Group) Corporation Limited, Tianjin 300301, China; 3 National Engineering Research Center for Equipment and Technology of Cold Strip Rolling, Yanshan University, Qinhuangdao 066004, China

**Keywords:** sulfide stress cracking (SSC), martensitic steel, tempering, double cantilever beam test, hydrogen permeation, SSC initiation, SSC propagation

## Abstract

A medium-carbon Cr–Mo–V martensitic steel was thermally processed by quenching (Q) at 890 °C and tempering (T) at increasing temperatures from 650 °C to 720 °C and the effect of tempering temperature, *T*_t_, on sulfide stress cracking (SSC) behaviors was estimated mainly via double cantilever beam (DCB) and electrochemical hydrogen permeation (EHP) tests and microstructure characterization. The results indicate that the threshold stress intensity factor for SSC, *K*_ISSC_, increased with increasing *T*_t_. The overall and local H concentration around the inclusions decreased with increasing *T*_t_, due to reductions in the amounts of solute atoms, grain boundaries and dislocations, which effectively prevented SSC initiation. Also, increasing *T*_t_ caused an increased fraction of high-angle boundaries, which evidently lowered the SSC propagation rate by more frequently diverting the propagating direction and accordingly restricted SSC propagation. The overall SSC resistance of this Q&T–treated steel was therefore significantly enhanced.

## 1. Introduction

Quenching and tempering (Q&T)–treated martensitic steels have long been widely used for important oil country tubular goods production, such as C90–C110 or higher grades for sour oil/gas well service [1,2], T125 and V150 grades for ultra-deep oil/gas well service [3,4,5] and 155 ksi or higher grades for perforation and drill services [6]. However, these high-strength martensitic steels normally have a high susceptibility to sulfide stress cracking (SSC)/hydrogen-induced cracking (HIC) [1]. Therefore, there have been extensive efforts [7,8,9,10,11,12,13,14,15,16,17,18,19,20,21,22] to understand the mechanisms governing SSC/HIC behaviors and to achieve the desired combination of high strength, high toughness and superior SSC/HIC resistance in these steels by optimizing the alloying design, metallurgical quality and Q&T parameters. 

Among these efforts, the correlations between various metallurgical factors and the susceptibility to SSC/HIC have recently received increasing attention [7,8,9,10,11,12,13,14,15,16,17,18,19,20,21,22]. Once these steels are exposed to the sour environment, atomic hydrogen (H) can form on the material surface due to the H^+^→H reaction [11], which depends on environmental and surface conditions. After the H atoms enter the material, these metallurgical factors might have their respective impacts on H mobility or diffusion [10], by acting as reversible (solute atoms, dislocations, grain boundaries, etc.) or irreversible (nonmetallic inclusions, precipitated particles, voids, etc.) traps for H atoms, depending on their binding enthalpies with H [12] and contributing to the susceptibility to SSC/HIC in different degrees [23,24,25]. Some investigations have suggested that reversible traps, where the H commonly escapes at room temperature, might have a strong effect on H embrittlement [13]. In contrast, irreversible traps, where H can escape only at high temperatures, such as 250 °C or above, might not participate in this behavior [13,14,15]. However, previous works have also identified SSC/HIC initiation at certain inclusions, such as non-deformable Al_2_O_3_ [17,18] and large-sized TiN/TiC particles [19] and at their interface with the matrix [26], even though they were irreversible traps. Moreover, once SSC/HIC initiates, it might propagate toward certain preferred directions or zones, such as the phase boundary, the segregation zone, or the particular crystallographic orientation. According to Reference [20], high-angle grain boundaries (HAGBs) with a high misorientation angle (MA ≥ 15°) might store higher energy and provide both more potent sites for H trapping and easier paths for crack propagation, in comparison with low-angle grain boundaries (LAGBs, MA < 15°), eventually leading to higher susceptibility to HIC in X70 pipeline steel. On the contrary, it was reported that for Q&T–treated martensitic steels, SSC could be arrested at HAGBs [26] and the threshold stress intensity factor for SSC, *K*_ISSC_, increased with decreasing size of martensitic packet with high-angle boundaries [22], indicating enhanced SSC resistance. On the other hand, Omura et al. [27] reported significantly enhanced SSC resistance in a martensitic steel via increases in tempering temperature, *T*_t_ and time and attributed it to a martensitic ferrite matrix with fewer dislocations and more dispersed carbides. This indicates that the SSC resistance of martensitic steels could be effectively enhanced by improving the tempering process. 

Therefore, these various metallurgical factors have exerted complex effects on the susceptibility to SSC/HIC, which were originally and essentially correlated to SSC evolution, including three stages of the entry of H atoms, SSC/HIC initiation and propagation. The effects of some metallurgical aspects (e.g., GB, dislocation and precipitate) on a single stage of SSC/HIC evolution in martensitic/bainitic steels, namely the entry of H atoms and SSC/HIC propagation, were estimated in previous work [28] by electrochemical hydrogen permeation (EHP) [29] and double cantilever beam (DCB) tests [30]. This led to some contradictory effects (e.g., GBs) on the overall resistance to SSC/HIC in previous reports. It was therefore essential to further clarify their effects on the overall resistance to SSC/HIC by determining their correlation with the whole SSC/HIC evolution. However, these efforts have been rarely reported, to the authors’ knowledge. 

For this attempt, a martensitic steel was thermally processed via Q&T at differing *T*_t_, providing a group of samples with quite different microstructural features of GBs, dislocations and precipitates. Their effects on SSC behavior, namely H mobility, SSC initiation/propagation and overall resistance to SSC (*K*_ISSC_), were estimated mainly through microstructure characterization and EHP and DCB tests. The mechanisms governing these correlations are also discussed.

## 2. Results

### 2.1. Mechanical Property and K_ISSC_ Values

The mechanical properties of each Q&T–treated sample are summarized in Table 1, indicating that the yield strength (*YS*), tensile strength (*TS*) and hardness (*HRC*) decreased, while the elongation (*EL*) at rupture increased with elevating *T*_t_. The *K*_ISSC_ for each Q&T–treated sample was also determined via the DCB test and is also summarized in Table 1. As the table shows, *K*_ISSC_ increased from 17.16 MPa∙mm^−0.5^ to 33.26 MPa∙mm^−0.5^ with increasing *T*_t_ from 650 °C to 720 °C, indicating an enhanced overall resistance to SSC due to the increased *T*_t_.

**Figure 1:**
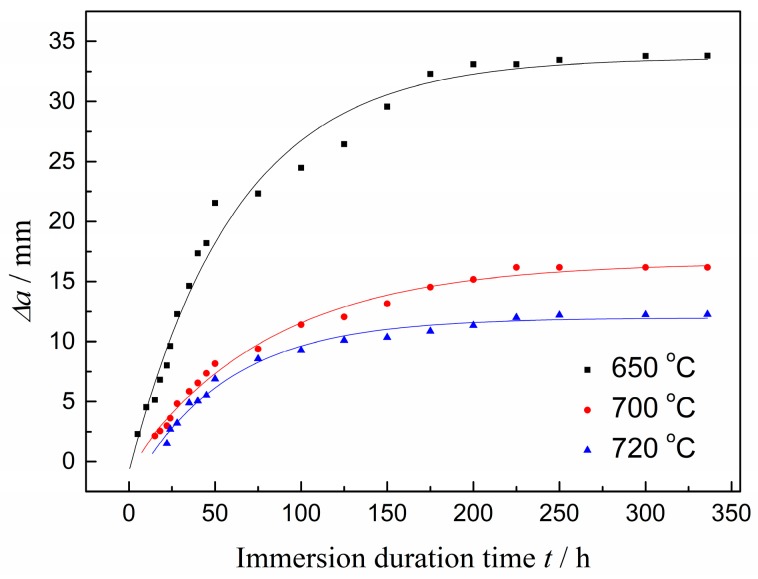
*Δa* as a function of immersion duration time (*t*) for Q&T–treated samples at differing *T*_t_.

The instantaneous SSC propagation length for each Q&T–treated sample was measured with the DCB test interrupted, according to the NACE (National Association of Corrosion Engineer) TM0177 standard [31] and evaluated as a function of immersing time (*t*), as shown in Figure 1. This reveals that SSC probably developed in three stages: initiation, rapid and palliative propagation. For the first stage, SSC was not detected for a couple of hours, the inoculation period after the DCB specimen was immersed in the solution, during which the diffusion of H atoms into the crystalline interstices and voids was assumed to be accumulated for initializing the H damage. For the second stage, SSC grew rapidly with the simultaneous assistance of the highly concentrated stress and the accumulated H atoms. For the third stage, SSC grew at a gradual reduced rate and approached a metastable state, where its propagation was almost imperceptible, probably due to the relaxation of concentrated stress and the approximately saturated H atom concentration.

As also indicated in Figure 1, the steel tempered at 720 °C had both the longest inoculation period for detected SSC and the least SSC propagation length among these Q&T–treated samples. SSC initiated over a longer time and propagated at a decreased length with the increased *T*_t_.

**Figure 2:**
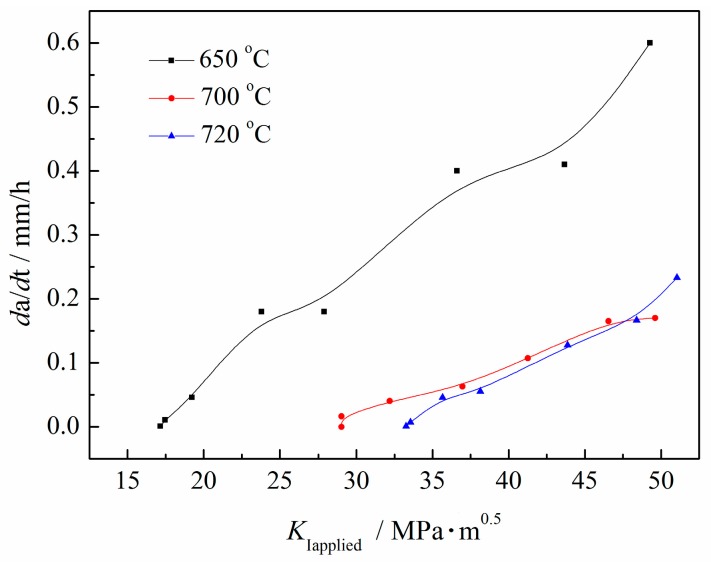
*da*/*dt* vs. *K*_Iapplied_ for Q&T–treated samples at differing *T*_t_.

SSC propagation behaviors were also interpreted in terms of the correlation between *da*/*dt* and *K*_Iapplied_, as shown in Figure 2. This indicates that *da*/*dt* decreased as a result of decreasing *K*_Iapplied_. With *K*_Iapplied_ decreased to a certain value, *da*/*dt* approached zero, where *K*_ISSC_ was obtained, according to the NACE TM0177 standard [31]. Furthermore, *da*/*dt* decreased and *K*_ISSC_ increased with increasing *T*_t_, indicating that the high-temperature tempering retarded SSC propagation and correspondingly enhanced SSC resistance.

### 2.2. SSC Fracture Morphology of the DCB Specimen

**Figure 3:**
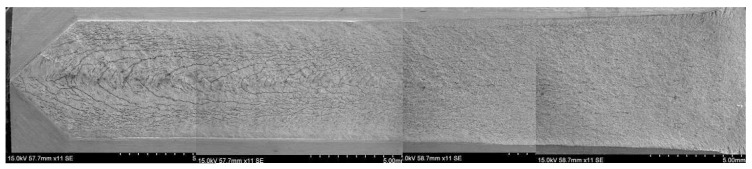
Typical macroscopic observation of sulfide stress cracking (SSC) fracture in double cantilever beam (DCB) sample tempered at 700 °C.

**Figure 4:**
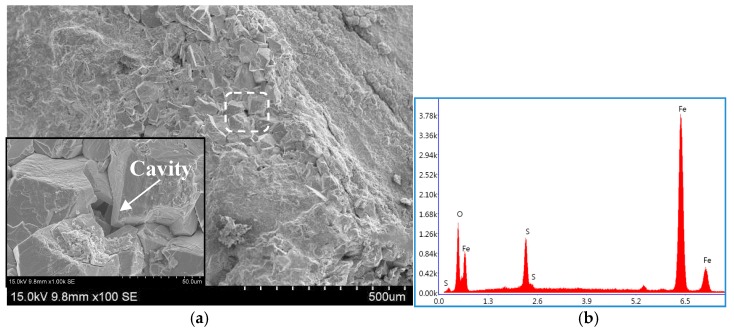
Typical SEM observation of corrosion film covering SSC fracture surface in (**a**) DCB sample and (**b**) its EDS (energy dispersive spectroscopy) spectrum.

Figure 3 shows a typical SSC fracture morphology of the DCB specimen tempered at 700 °C. It can be seen that the fracture surface was covered with a corrosion product film that was rich in Fe and S, as examined by EDS (energy dispersive spectroscopy) analysis, illustrated in Figure 4. The corrosion film was loose and featured microcracks and micropores, probably leading to poor resistance to the migration of HS^−^ or S^2−^ to the fresh material surface. 

**Figure 5:**
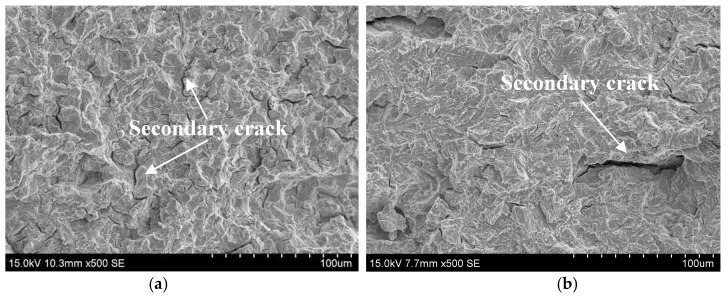

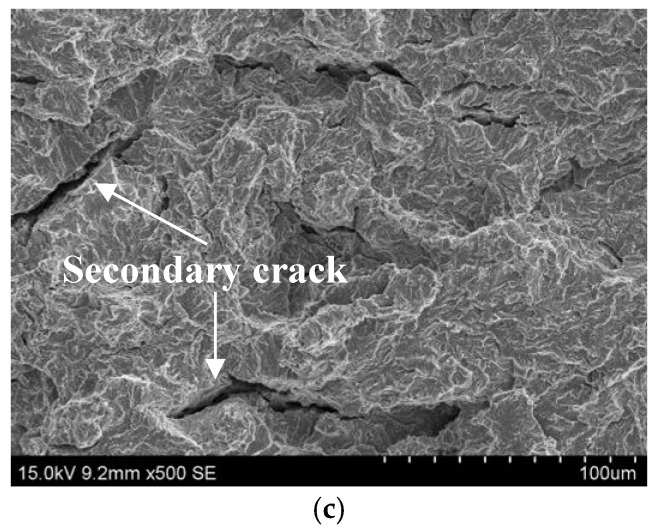
Typical SEM observations of real SSC fracture surface in DCB sample tempered at (**a**) 650; (**b**) 700; and (**c**) 720 °C, with the corrosion film removed.

The corrosion film was subsequently removed with a solution of dilute hydrochloric acid and hexamethylene tetramine and the real SSC fracture surface is shown in Figure 5. Actually, the SSC fracture was a typical quasi-cleavage cracking, featuring some random secondary cracks underneath the fracture surface, consistent with an early work reported by Dadfarnia et al. [28]. In addition, some intergranular fracture areas were also perceptible on the fracture surface in all the DCB samples.

### 2.3. Microstructure Observation

#### 2.3.1. Prior Austenite Grain Observation

**Figure 6:**
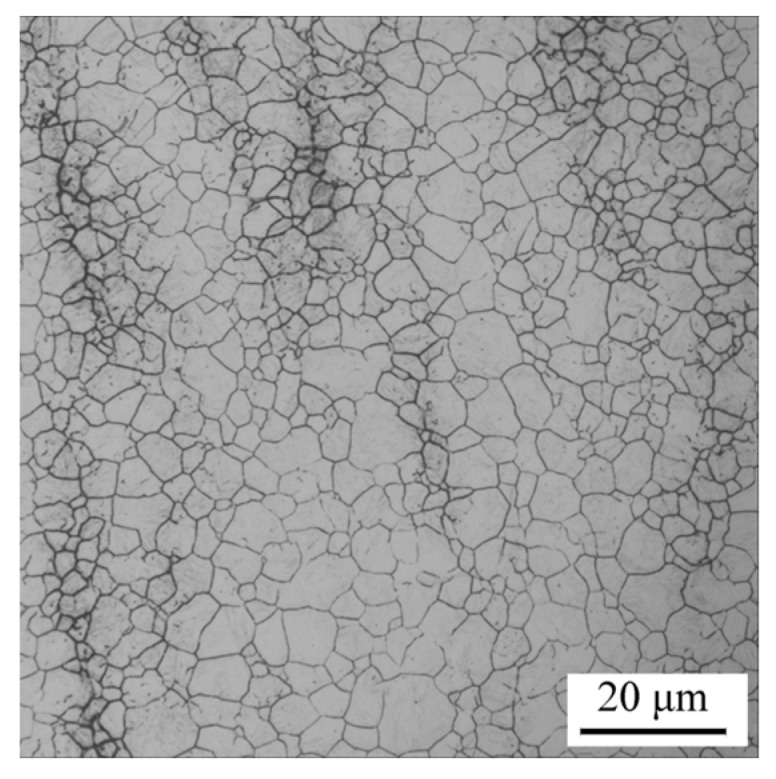
Typical prior austenite grain (PAG) observation of as-quenched sample at 890 °C.

The typical prior austenite grain (PAG) morphology of the samples quenched at an identical temperature of 890 °C, waiting for further tempering at differing *T*_t_, is shown in Figure 6. The average PAG size, *D*_γ_, was quantitatively estimated and is summarized in Table 2. As the table states, *D*_γ_ remained at almost the same level of 7.5 μm under identical quenching conditions. 

#### 2.3.2. Martensitic Structure Observations

**Figure 7:**
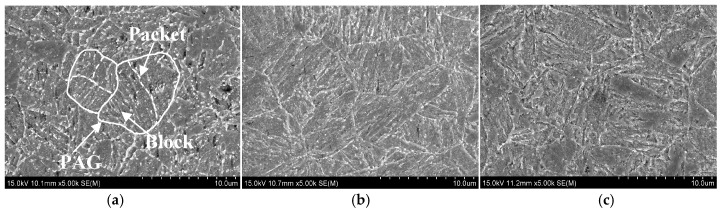
Typical SEM observations of martensitic packet in Q&T–treated samples at differing *T*_t_ of (**a**) 650; (**b**) 700; and (**c**) 720 °C.

**Figure 8:**
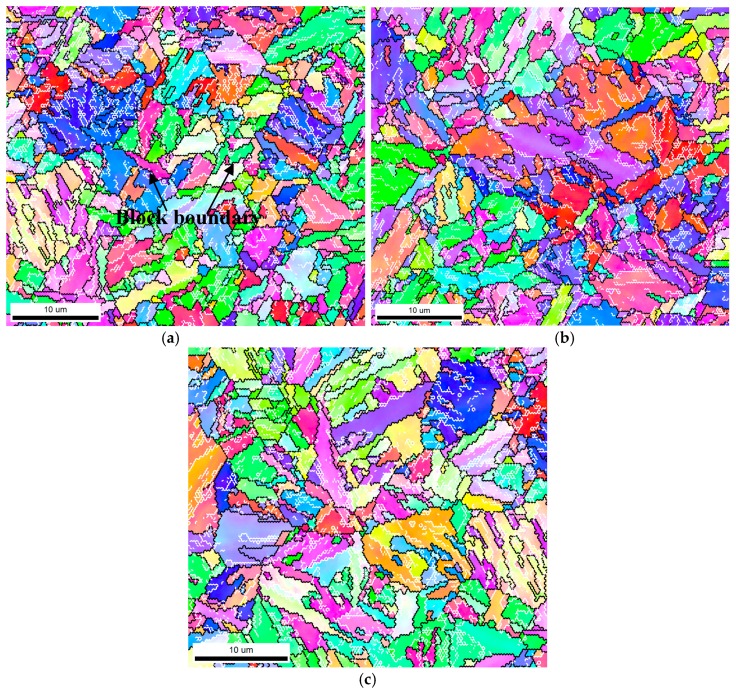
Typical electron back-scattered diffraction (EBSD) orientation maps showing martensitic packet and block in Q&T–treated samples at differing *T*_t_ of (**a**) 650; (**b**) 700 and (**c**) 720 °C.

The PAG of martensitic steel can normally be partitioned into three types of martensitic substructures: packet, block and lath [32,33]. The martensitic packet and block in each Q&T–treated sample were also characterized via SEM and electron back-scattered diffraction (EBSD), as shown in Figure 7 and Figure 8, respectively. The average packet and block size, *D*_p_ and *D*_b_, were quantitatively determined and are summarized in Table 2. This demonstrates that *D*_p_ and *D*_b_ increased with increasing *T*_t_ from 650 °C to 720 °C. The grain boundary misorientation angle (GBMA) might play a significant role in controlling SSC resistance by affecting hydrogen permeability [20,34] and crack propagation [21,26]. Therefore, the GBMA distribution of martensitic substructure in each Q&T–treated sample was also analyzed via EBSD and the fraction of the HAGBs, *f*_GBMA≥15°_, is summarized in Table 2. As presented, HAGBs were in the majority in each sample and *f*_GBMA≥15°_ increased slightly with increasing *T*_t_ from 650 to 720 °C.

#### 2.3.3. Martensitic Lath and Precipitate Observations

**Figure 9:**
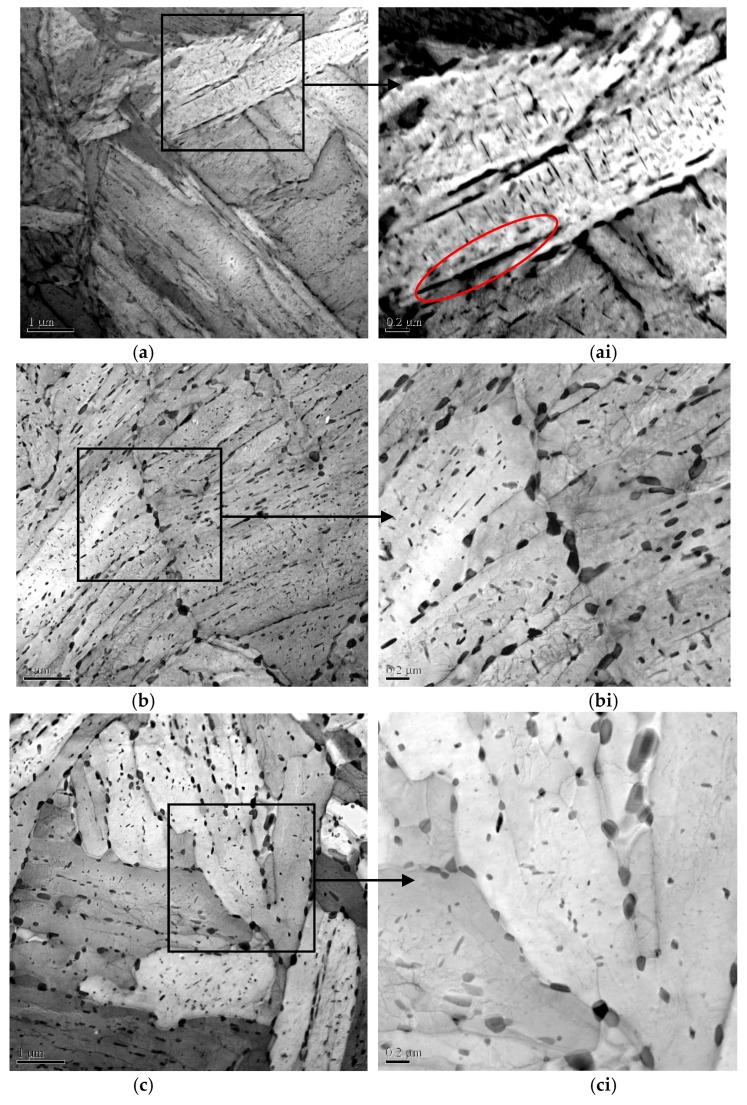
Typical TEM observations of martensite lath and precipitate in Q&T–treated samples at differing *T*_t_ of (**a**) 650; (**b**) 700; and (**c**) 720 °C and their enlarged micrographs (**ai**, **bi** and **ci**). The red frame showing the rod-like precipitated particles distributed in the martensitic laths boundaries.

TEM micrographs of each Q&T–treated sample are presented in Figure 9. As the figure shows, the martensite consisted of regular laths in the minority and irregular ones in the majority. The irregular laths had a curved boundary, which were assumed to derive from the polygonization of prior regular laths in as-quenched samples, due to tempering at a high *T*_t_. In contrast, a small quantity of regular laths might possess a particular crystallographic orientation with high thermodynamic stability [35] and could survive heavy tempering. Also, as this figure shows, the polygonized irregular martensitic laths evidently increased with elevating *T*_t_, at the expense of the regular martensitic laths. The average lath width, *W*_l_, was further determined quantitatively and is also summarized in Table 2. *W*_l_ obviously increased with increasing *T*_t_, indicating a widening of the martensitic lath, probably due to the interface migration or the merging of two neighboring laths [36].

**Figure 10:**
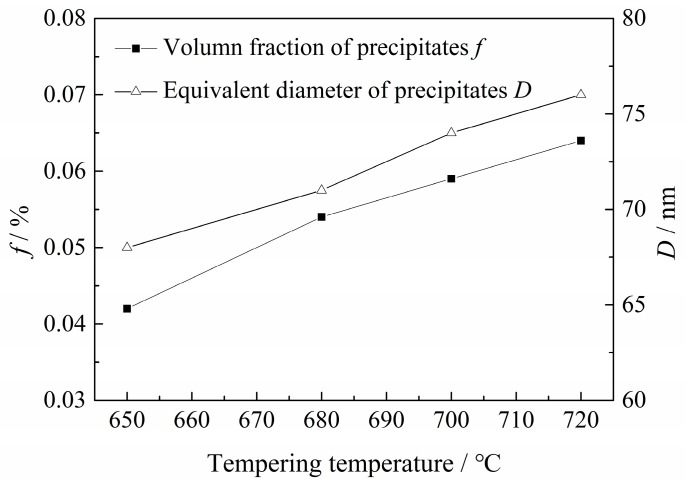
*D* and *f* of the precipitates in Q&T–treated samples varied with *T*_t_.

There still appeared to be a large quantity of rod-like and spherical precipitated particles distributed in the martensitic laths or at their boundaries, as also shown in Figure 9. Meanwhile, the rod-like precipitated particles were spheroidized gradually as the *T*_t_ increased. The average equivalent diameter, *D* and the volume fraction, *f*, of the precipitates were further estimated quantitatively and are provided as the function of *T*_t_ in Figure 10. This shows that both the *D* and *f* of precipitates obviously increased with increasing *T*_t_.

#### 2.3.4. Dislocation Estimations

**Figure 11:**
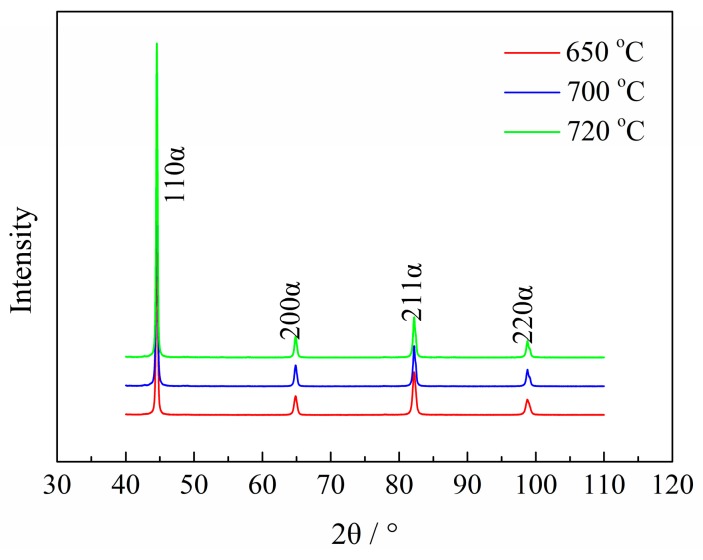
XRD patterns of Q&T–treated samples at differing *T*_t_.

The dislocation density, *ρ*, of each Q&T–treated sample was determined by XRD pattern, as shown in Figure 11 and summarized in Table 2. The dislocation density decreased from 1.41 × 10^14^ to 0.62 × 10^14^ m^−2^ with increasing *T*_t_ from 650 °C to 720 °C. The present result is in agreement with a previous work by Masakatsu et al. [37]. They reported that the half-width of the (211) peak in the XRD pattern, indicating the dislocation density, reduced with elevated *T*_t_ due to the addition of 0.1 wt % vanadium to the 0.5Cr–0.7Mo steel. 

### 2.4. Hydrogen Permeation

**Figure 12:**
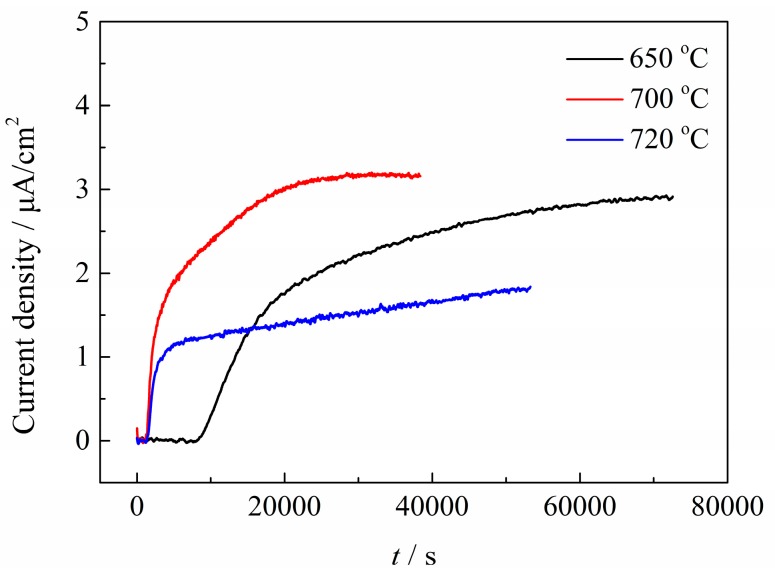
H permeation curves for Q&T–treated samples at differing *T*_t_.

Figure 12 shows the H permeation curves for Q&T–treated samples. The H diffusion coefficient, *D*_0_ and the diffusion H concentration, *C*_0_, were calculated with Equations (10) and (11) and the results are summarized in Table 3. As the table shows, *D*_0_ increased and *C*_0_ decreased with increasing *T*_t_, indicating lower H permeability.

## 3. Discussion

### 3.1. Relationships among Tempering Temperature, H Mobility and SSC Initiation

#### 3.1.1. Effect of Tempering Temperature on H Permeability

*C*_0_, indicating H permeability, decreased with increasing *T*_t_, as shown in Figure 12 and Table 3. Actually, *C*_0_ is dominantly dependent on the density of reversible H traps, *N*_T_, on the assumption that the irreversible traps could be saturated by the H atoms [38]. According to previous works [39,40,41,42], H traps such as solute atoms, GBs and dislocations were generally regarded as reversible traps, due to their bonding energy being lower than 60 kJ/mol. Among these reversible traps, the solute atoms were assumed to decrease with increasing *T*_t_, due to increased precipitation, as shown in Figure 9 and Figure 10. Accordingly, the number of reversible traps provided by the solute atoms, *N*_T-SA_, also decreased with increasing *T*_t_. However, the precipitates in this Q&T–treated Cr–Mo–V–Ti–B martensitic steel were highly diversified and complicated in their structure [43] and hence it is rather intricate to discern between the atoms consumed for these precipitates and the residual solute atoms. Quantification of *N*_T-SA_ therefore remains challenging. Accordingly, the *N*_T_ for these Q&T–treated samples at differing *T*_t_ were quantified mainly as functions of the GBs (PAG, martensitic packet/block/lath boundaries, etc.) and the dislocations, respectively, to better understand their effects on *C*_0_.

The H trap density provided by the GBs, *N*_T-GB_, was estimated, with the grain assumed to be an approximate sphere and counted as *n* per cubic meter, using the following equation [44]:

(1) $$N_{T\text{-}GB}=\frac{nS_{G}x_{GB}}{2nV_{G}x_{a}{}^{3}}$$

where *V*_G_ and *S*_G_ are the volume and surface area of one grain, respectively; the factor of 1/2 is due to one grain plane shared by two grains; *x*_a_ is atomic distance, 0.26 nm; *x*_GB_ is the width of GB, about 10 times that of *x*_a_. Equation (1) can then be simplified as:

(2) $$N_{T\text{-}GB}=\frac{30}{d_{G}x_{a}{}^{2}}$$

where *d*_G_ is the grain size. This equation indicates that the *N*_T-GB_ is in inverse proportion to *d*_G_, by which *N*_T-GB_ for these Q&T–treated samples were estimated and are listed in Table 4. 

The H trap density provided by the dislocations, *N*_T-dis_, was estimated by:

(3) $$N_{T\text{-}\mathrm{dis}}=\frac{5\rho_{\mathrm{dis}}}{x_{a}}$$

where *ρ*_dis_ is dislocation density and the factor of 5 is assumed to be the ratio of the dislocation width to *x*_a_. The *N*_T-dis_ for these Q&T–treated samples were calculated and are also listed in Table 4.

As Table 4 shows, the estimated *N*_T-MP_, *N*_T-MB_ and *N*_T-ML_ provided by the martensitic packet, block and lath boundaries decreased with their increasing size and accordingly their decreasing number of boundaries, whereas *N*_T-PAG_ remained at almost the same level. As the martensitic lath boundaries normally occupied all the PAG and the martensitic packet/block boundaries, *N*_T-ML_ contained *N*_T-PAG_, *N*_T-MP_ and *N*_T-MB_. In general, the PAG and the martensitic packet/block boundaries were confirmed to be HAGBs [45] and correspondingly the effective reversible traps [34], whereas the lath boundaries in as-quenched martensitic steels were normally accepted as LAGBs with MA ≤ 10° [32], probably contributing little to the *C*_0_ [34]. However, according to the EBSD analyses in Figure 8 and the actual TEM observations in Figure 9, the HAGBs surrounding the irregular laths constituted the major fraction of the martensitic lath boundaries due to their polygonization via heavy tempering, essentially supplying much to the *C*_0_. In contrast, the remnant LAGBs encircling the regular laths comprised a minor fraction of the martensitic lath boundaries, still contributing little to the *C*_0_. Thus, the final *N*_T-GB_ was calculated approximately by combining the *N*_T-ML_ (in Table 4) and the fraction of HAGB, *f*_GBMA≥15°_, essentially for the polygonized irregular martensitic lath (in Table 2), that is, *N*_T-GB_ = *N*_T-ML_ × *f*_GBMA≥15°_, with the result presented in Table 4. 

Consequently, the amount of reversible H traps provided by the various GBs, the dislocations and even the solute atoms all decreased with increasing *T*_t_ and therefore the overall diffusion H concentration (*C*_0_) decreased markedly, as shown in Table 3.

#### 3.1.2. Effect of H Permeability on SSC Initiation

**Figure 13:**
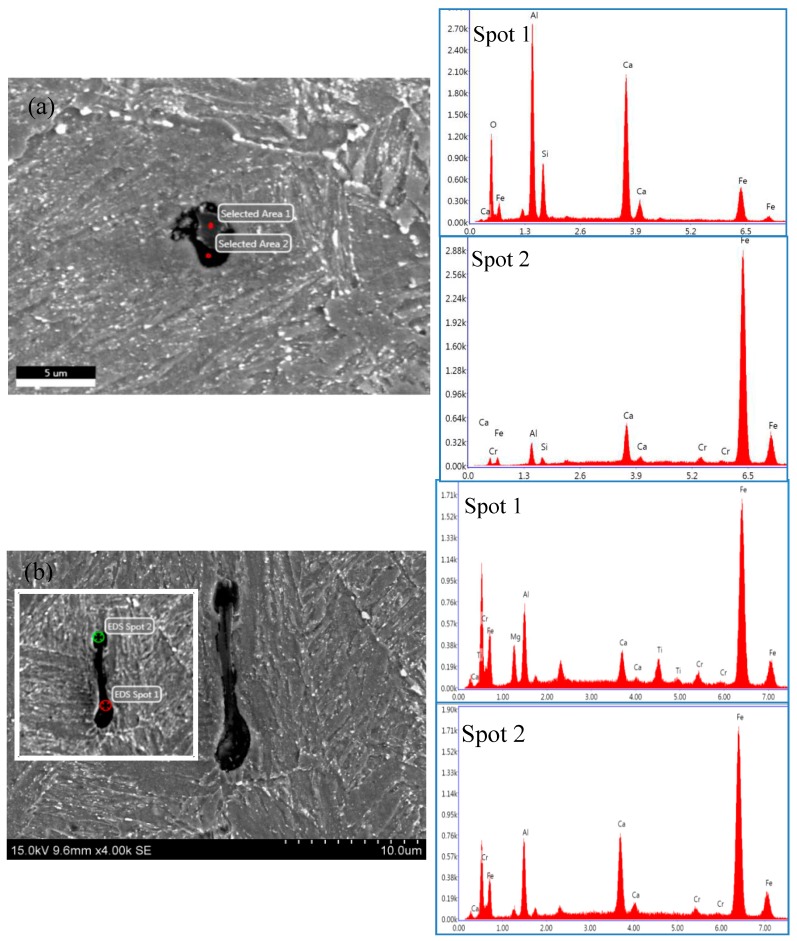
(**a**) Corrosion pit and (**b**) secondary microcrack typically initiating from massive Al_2_O_3_·SiO·CaO and slender Al_2_O_3_·SiO·CaO·TiO complex inclusions, respectively, underneath the SSC fracture surface of DCB sample tempered at 700 °C.

A certain number of inclusions with a large size of more than 3 µm were still present in this experimental steel, although it was prepared by melting in an electric furnace and purifying in a vacuum degassing furnace. There appeared mainly two types of complex inclusions in each DCB sample: the massive Al_2_O_3_·SiO·CaO and the slender Al_2_O_3_·CaO·TiO particles, as shown in Figure 13a,b. The slender one was typically located in a corrosion pit where SSC was initiating. This observation was in agreement with previous work by Schiapparelli et al., in which HIC was triggered at a non-deformable inclusion of Al_2_O_3_ [17]. 

In addition, the local H concentration, *C*_σ_, of the highly stressed regions in the DCB sample can be estimated using the following equation [46]:

(4) $$C_{\sigma }=C_{0}exp\left(\sigma_{h}V_{H}/RT\right)$$

where *C*_0_ is the diffusion H concentration, *σ*_h_ is the hydrostatic stress, *V*_H_ is the partial molar volume of H in iron (2.1 × 10^−6^ m^3^/mol), *R* is the gas constant and *T* is the ambient temperature. As this equation indicates, *C*_σ_ decreased as the result of decreased *C*_0_. When the H-induced stress concentration exceeded the atomic binding force, SSC eventually initiated. 

Susceptibility to SSC initiation can be estimated by the incubation time for SSC origination, *t*_i_, which was attained by fitting the crack propagation length (*Δa*) vs. the time curve, as demonstrated in Figure 1. The optimum imitated Equations (5)–(7) for the samples tempered at 650, 700 and 720 °C, respectively, as follows:

(5) $$\Delta a=33.6582-34.4650\times 0.9841^{t}$$

(6) $$\Delta a=16.5937-17.0433\times 0.9879^{t}$$

(7) $$\Delta a=11.9662-14.4518\times 0.9820^{t}$$

Supposing *Δa* = 0, the *t*_i_ for the Q&T–treated samples, tempered at 650, 700 and 720 °C was 1.5, 2.2 and 10.4 h, respectively, indicating a lowered susceptibility to SSC initiation with increasing *T*_t_. 

Therefore, the increasing *T*_t_ led mainly to martensitic lath polygonization and widening, providing a decreased amount of HAGBs and accordingly reversible H traps, inducing a lowered overall and local diffusion H concentration (*C*_0_ and *C*_σ_), leading to a depressed stress concentration around the inclusions and simultaneously a prolonged incubation time and finally decreased susceptibility to SSC initiation.

### 3.2. Effect of GBs on SSC Propagation

**Figure 14:**
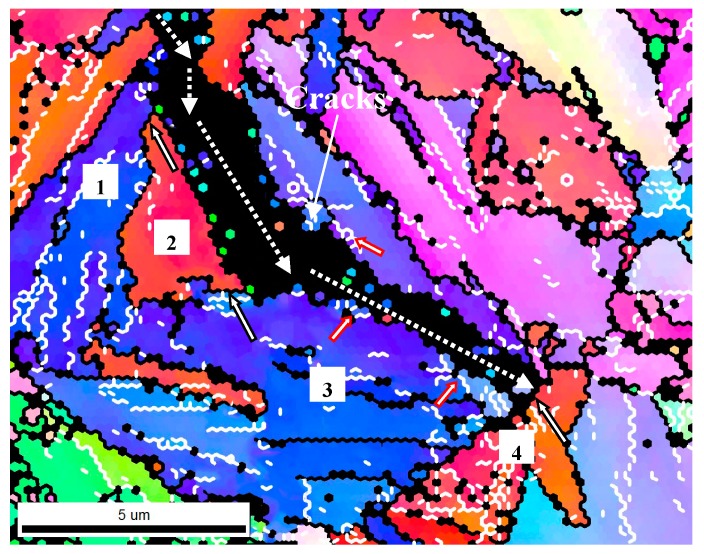
Typical inverse pole figure map displaying grain boundaries with different crystal orientations in a Q&T–treated sample at *T*_t_ = 700 °C. Dotted arrow line stands for the crack propagation direction; black solid arrow line denotes high-angle grain boundaries (HAGBs) and red solid arrow lines indicate low-angle grain boundaries (LAGBs).

To further understand the effect of GBs with certain crystallographic orientations on crack propagation, secondary microcracks underneath the SSC fracture surface of DCB samples were observed and the corresponding misorientation angles between grains adjacent to the crack were analyzed by EBSD, as shown in Figure 14 and Table 5. LAGB/HAGB is generally defined as the grain boundary with a misorientation angle of 2–15°/>15° [47], indicated by the white/black line. It can be seen that the microcrack propagated unimpededly through the LAGBs within grains 1, 2 and 3, indicating that the LAGBs could not hinder the SSC from propagating. In contrast, the microcracks diverted direction when they came across the HAGBs of grains 2 and 3 and finally arrested also at the HAGBs of grains 3 and 4. 

Hence, HAGB could play a significant role in hindering SSC propagation, whereas LAGB failed to provide an evident barrier, showing the two different impacts on crack propagation. According to previous observations [48], the change in operating slip system was essential for the GB to act as a barrier for the dislocation movement when the crack came across the GB. For the HAGB, its own operated slip system might induce a dislocation-free zone formation in the adjacent grain and accordingly lead to a significantly diverted direction of the crack. In contrast, LAGB could only yield a relatively low retardation of crack propagation, due to the minor difference in orientation between the two neighboring grains. 

The increasing *T*_t_ brought about martensitic lath polygonization and widening to a higher degree, providing an increased fraction of HAGBs and also an increased mean GBMA, restraining SSC propagation by lowering their propagation rate (Figure 2), finally resulting in an elevated *K*_ISSC_ value.

## 4. Materials and Methods

### 4.1. Materials and Heat Treatment

The 28CrMo48VTiB martensitic steel used for this work was melted in an electric furnace, continuously cast with argon shielding and thermally pieced-rolled-stretched into a seamless tube 177.8 × 10.36 mm (diameter × thickness). The chemical compositions are presented in Table 6.

The samples with dimension 300 × 100 mm were cut from the steel tube, reheated at 890 °C for 20 min and water quenched and consequently tempered at differing *T*_t_ of 650, 700 and 720 °C for 60 min.

### 4.2. Estimations of Mechanical Properties and SSC Susceptibility

**Figure 15:**
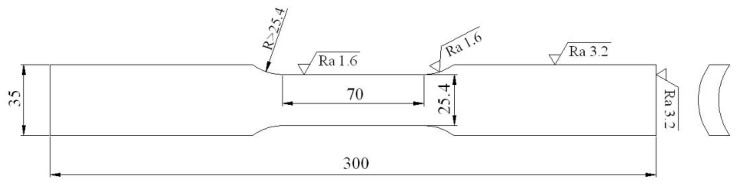
Geometry and dimensions of the tensile specimen.

The conventional tensile properties of each Q&T–treated sample were determined according to the ASTM (American Society for Testing Material) Standard A370-10 [49]. Each sample was machined along the longitudinal direction of the tube, with geometry and dimensions as shown in Figure 15 and tensioned in a CMT5305 Instron machine at room temperature. Two tensions for each sample were performed and their average values are reported. The hardness for each sample was also determined in a Rockwell hardness tester using a load of 1500 N. For precision, the hardness at 6 different positions in each sample were measured and their average values are reported.

**Figure 16:**
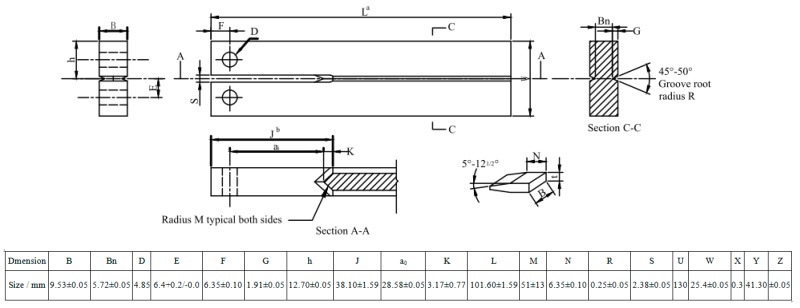
Typical illustration of standard DCB specimen.

The susceptibility to SSC in each sample was estimated with the DCB test, according to the NACE TM0177 standard, Method D [31]. The standard DCB specimen is illustrated in Figure 16. A double-taper wedge with certain thickness was selected to provide the desired arm displacement and load by inserting into the DCB specimen. All DCB tests were performed with their specimens immersed in the same standard NACE TM0177 solution A, consisting of 5.0 wt % NaCl and 0.5 wt % CH_3_COOH dissolved in distilled water saturated with 1 atm H_2_S. The pH value of the solution did not exceed 3 at the start and 4 at the end of the test. The tests were performed for 2 weeks at ambient temperature, during which the instantaneous applied stress intensity factor, *K*_Iapplied_ and the threshold stress intensity factor, *K*_ISSC_, were obtained using Equations (8) and (9):

(8) $$K_{\mathrm{Iapplied}}=\frac{p\times a_{i}(2\sqrt{3}+2.38h/a_{i}){\left(B/B_{n}\right)}^{1/\sqrt{3}}}{B\cdot h^{1.5}}\frac{\left(-26.232+51.886a/h+8.523{\left(a/h\right)}^{2}+8.5178{\left(a/h\right)}^{3}\right)}{\left(-26.232+51.886a_{i}/h+8.523{\left(a_{i}/h\right)}^{2}+8.5178{\left(a_{i}/h\right)}^{3}\right)}$$

(9) $$K_{\mathrm{ISSC}}=\frac{p\times a(2\sqrt{3}+2.38h/a){\left(B/B_{n}\right)}^{1/\sqrt{3}}}{B\cdot h^{1.5}}$$

where *P* is the final equilibrium wedge lift off load, determined from an abrupt change in the slope of load-displacement curve; *a*_i_/*a* is the instant/final crack length of SSC for estimating *K*_Iapplied_/*K*_ISSC_, obtained by subtracting 6.35 mm from the distance between the slot end and the mean position of the crack front; *h* is the arm height; and *B* and *B*_n_ are the DCB specimen and web thickness, respectively. Three parallel DCB tests were performed for each Q&T–treated sample and the average values are reported.

In order to further understand SSC crack initiation and propagation behaviors occurring in the DCB tests and their dependence on metallurgical aspects, the crack propagation length, *Δa*, as a function of immersing time, *t*, was measured by the NACE TM0177 standard, Method D [31]. Then the crack propagation rate, *da*/*dt*, was obtained by seeking derivation of the *Δa* vs. *t* curves. Finally, *da*/*dt* as a function of the corresponding *K*_Iapplied_ was described.

### 4.3. Electrochemical H Permeation Test

The H permeation behavior in each Q&T–treated sample was quantitatively estimated with the EHP test at 298 K using the classical Devanathan-Stachursky technique. The electrolyte for H charging was a solution of 0.5 mol/L H_2_SO_4_ and 0.25 g/L CH_4_N_2_S, while the electrolyte for H expanding was an aqueous solution of 0.2 mol/L NaOH. The H charging time might depend on the purpose. The sample for the H permeation estimation was 0.8 mm thick, with the nickel-plated surface acting as the anode side. The current was measured when the background current decreased and reached a steady value of 0.1 μm/cm^2^ or lower.

The diffusion coefficient of H, *D*_0_ (cm^2^/s), was calculated using the following equation:

(10) $$D_{0}=\frac{L^{2}}{6t_{0.63}}$$

where *L* is the specimen thickness (cm) and *t*_0.63_ is the time lag for 0.63 saturation of permeation current density at the steady state (s). The reversible H concentration, *C*_0_ (ppm), was estimated using the following equation:

(11) $$C_{0}=\frac{i_{\infty }L}{FD_{0}}$$

where *i*_∞_ is the steady-state atomic H permeation current, μA/cm^2^ and *F* is Faraday’s constant, 9.6485 × 10^10^ μA·s/mol.

### 4.4. Microstructure Observation

The as-quenched sample at 890 °C was etched in a supersaturated picric acid aqueous solution for optical microscopy (OM) observations of the prior austenite grain (PAG). Each Q&T–treated sample was etched in 4% nital for martensitic packet observation in a Hitachi S-3400N scanning electron microscope (SEM). The electron back-scattered diffraction (EBSD) map was obtained by a SUPRA550 field emission scanning electron microscope equipped with a TSL (Tinysoft Statistical analysis Language) orientation imaging microscopy system to reveal the martensitic block substructure and the misorientation angles between the adjacent martensitic substructure boundaries. SSC inducing the fractured surface and the secondary crack in a cross-section of the DCB specimen after each trial were also examined by SEM and EBSD, respectively. The crystallography orientation information of the martensitic packet/block structures next to the secondary crack and their correlations were determined; Channel 5 software from Oxford-HKL was used for the orientation data processing. Each EBSD sample was polished using 0.04 µm colloidal silica slurry in a vibratory polisher for 1 h. The characteristics of the martensitic lath and the distribution, shape and chemical compositions of precipitated particles were determined by examining thin foils or carbon extraction replicas using a JEM-2010 high-resolution transmission electron microscope.

The martensitic structure size for each Q&T–treated sample was quantitatively estimated by averaging at least 200 PAGs, 200 packets, 150 blocks and 100 laths from the OM, SEM, EBSD and TEM micrographs, respectively. For estimating the equivalent diameter (*D*_o_) and volume fraction (*f*) of precipitated particles of different shapes, the rod-like particles were considered with the spherical particles and *D*_o_ was regarded as the particle size. Then *f* was calculated with the following equation:

(12) $$f=\frac{N\frac{4\pi }{3}}{S_{0}D_{o}}{\left(\frac{{}_{D_{o}}}{2}\right)}^{3}=\frac{N\pi D_{o}^{2}}{6S_{0}}=\frac{2NS}{3S_{0}}$$

where *N* is the number of particles per area, *S*_0_ is the specific area for estimation and *S* is the particle area.

The dislocation density of each Q&T–treated sample was determined by quantitative X-ray diffraction (XRD) analysis. XRD spectra were obtained by scanning over a scanning angle (2*θ*) range of 40–110° and at a step size of 0.02° in a Rigaku D/max-2500/PC diffractometer (Rigaku, Tokyo, Japan). In order to correct the XRD line broadening resulted from the instrument, the XRD pattern of a standard sample of Si powder which was annealed at 300 °C for 24 h was measured. This diffraction line broadening correction was performed with the data processing via the software of Jade 5 (5.0, Jade Software, Christchurch, New Zealand).

## 5. Conclusions

The effect of increasing tempering temperature (*T*_t_) on SSC behavior in a Q&T–treated medium carbon Cr–Mo–V martensitic steel was estimated in this work and the conclusions are summarized as follows:

1. *K*_ISSC_ and the overall resistance to SSC of this martensitic steel were obviously enhanced by increasing *T*_t_ from 650 to 720 °C.
2. Increased *T*_t_ led to decreases in overall and local H concentration around the inclusions, owing to martensitic lath polygonization and widening and resulted in a lowered susceptibility to SSC initiation.
3. Increased *T*_t_ brought about a higher fraction of HAGBs and resulted in enhanced resistance to SSC propagation by more frequently diverting the propagating direction and lowering the propagation rate.

## Figures and Tables

**Table 1:** Summary of mechanical properties and *K*_ISSC_ value for quenching and tempering (Q&T)–treated samples. *YS*: yield strength; *TS*: tensile strength; *EL*: elongation; *HRC*: hardness.

Sample	*T*_t_/°C	*YS*/MPa	*TS*/MPa	*EL*/%	*HRC*	*K_ISSC_*/MPa·mm^0.5^
1	650	1007	1062	18	35.4	17.16
2	700	776	857	23	25.1	29.02
3	720	728	805	25	23.4	33.26

**Table 2:** Quantitative determinations of PAG and martensitic substructure features in Q&T–treated samples at differing *T*_t_.

*T*_t_/°C	*D*_γ_/μm	*D*_p_/μm	*D*_b_/μm	*W*_l_/μm	*f*_GBMA≥15°_/%	*ρ*/10^14^ m^−2^
650	7.6	3.73	1.15	0.32	66.8	1.41
700	7.5	4.01	1.32	0.53	69.8	0.84
720	7.1	4.20	1.35	0.58	70.5	0.62

**Table 3:** H permeation test results for Q&T–treated samples at differing *T*_t_.

*T*_t_/°C	*D*_o_/10^−7^cm^2^/s	*C*_o_/ppm
650	0.48	6.45
700	1.78	1.87
720	2.20	0.86

**Table 4:** Summary of calculated *N*_T_ provided by various grain boundaries (GBs) and the dislocations.

*T*_t_/°C	650	700	720
*N*_T-PAG_/10^25^ m^−3^	5.8	5.9	6.2
*N*_T-MP_/10^25^ m^−3^	11.9	11.1	10.6
*N*_T-MB_/10^25^ m^−3^	38.6	33.6	32.9
*N*_T-ML_/10^25^ m^−3^	138.7	83.7	76.5
*N*_T-GB_/10^25^ m^−3^	92.6	58.4	53.9
*N*_T-dis_/10^25^ m^−3^	0.27	0.16	0.12

**Table 5:** Effects of HAGBs on SSC propagation behavior shown in Figure 14.

Grain Boundary	Misorientation Angle (°)	Cracking Behavior
1 and 2	49.3	Deviated
2 and 3	35.6	Deviated
3 and 4	59.5	Arrested

**Table 6:** Chemical compositions of 28CrMo48VTiB steel (wt %).

C	Si	Mn	P	S	Cr	Mo	V	Ti	B
0.28	0.25	0.40	0.008	0.002	1.00	0.80	0.15	0.015	trace
